# Stem cell-based drug delivery strategy for skin regeneration and wound healing: potential clinical applications

**DOI:** 10.1186/s41232-023-00287-1

**Published:** 2023-06-30

**Authors:** Weiyue Zhang, Xin Huang

**Affiliations:** 1 Department of Endocrinology, Union Hospital, Tongji Medical College, Huazhong University of Science and Technology, Wuhan, 430022 China; 2 Department of Orthopaedics, Union Hospital, Tongji Medical College, Huazhong University of Science and Technology, Wuhan, 430022 China

**Keywords:** Stem cell, Drug delivery strategy, Cell membrane-coating nanotechnology, Skin regeneration, Wound healing

## Abstract

Stem cell-based therapy is widely accepted to be a promising strategy in tissue regenerative medicine. Nevertheless, there are several obstacles to applying stem cells in skin regeneration and wound healing, which includes determining the optimum source, the processing and administration methods of stem cells, and the survival and functions of stem cells in wound sites. Owing to the limitations of applying stem cells directly, this review aims to discuss several stem cell-based drug delivery strategies in skin regeneration and wound healing and their potential clinical applications. We introduced diverse types of stem cells and their roles in wound repair. Moreover, the stem cell-based drug delivery systems including stem cell membrane-coated nanoparticles, stem cell-derived extracellular vesicles, stem cell as drug carriers, scaffold-free stem cell sheets, and stem cell-laden scaffolds were further investigated in the field of skin regeneration and wound healing. More importantly, stem cell membrane-coating nanotechnology confers great advantages compared to other drug delivery systems in a broad field of biomedical contexts. Taken together, the stem cell-based drug delivery strategy holds great promise for treating skin regeneration and wound healing.

## Introduction

The skin is widely known to be the largest organ in humans, which could maintain homeostasis and protect internal organs. Wounds, especially chronic wounds, burn wounds, or infective wounds, are often accompanied by delayed recovery and result in immense financial burdens to society. Besides, the rapidly growing population of patients with diabetes or obesity contributes to the increased prevalence of chronic wounds. About 15% of diabetic patients suffer from diabetic foot ulcers and about 84% of cases of amputation of the lower part of the foot [1]. The skin is mainly made of three layers: epidermis, dermis, and hypodermis [2]. Mechanically, the processes of wound healing mainly consist of three overlapping but distinct stages including inflammation, proliferation, and remodeling, which are modulated by a great number of growth factors, cytokines, and chemokines [3]. Furthermore, the dysregulations of cellular and molecular signals during the above stages might finally lead to chronic wounds [4]. Although growing investigations have been focused on promoting cutaneous wound healing, in-depth management and novel strategies for skin regeneration and wound healing remain in urgent need.

Stem cell-based therapy is widely regarded as a promising strategy in tissue regenerative medicine. Stem cells are self-renewable and can differentiate into diverse types of cells, which is important for physiologic tissue regeneration after injury [5]. Stem cell-based therapy might not only accelerate earlier wound closure and skin regeneration but also prevent wound contracture and scar formation [6]. Previous studies have investigated the roles of stem cells in skin regeneration and wound repair and methods of improving the therapeutic effects of stem cells [6–8]. Nevertheless, there remain several obstacles to applying stem cells in skin regeneration and wound healing, including determining the optimum source, the processing and administration methods of stem cells, and the survival of stem cells in wound sites [9].

Owing to the limitations of applying stem cells in skin regeneration and wound healing directly, this review aims to discuss several stem cell-based drug delivery strategies in skin regeneration and wound healing and their potential clinical applications. We first introduced the various types of stem cells and their roles in wound repair. Moreover, the stem cell-based drug delivery systems including stem cell membrane-coated nanoparticles [10], stem cell-derived extracellular vesicles [11], stem cell as drug carriers [12], scaffold-free stem cell sheets [13], and stem cell-laden scaffolds [14] were further investigated in the field of skin regeneration and wound healing. More importantly, stem cell membrane-coating nanotechnology confers great advantages compared to other drug delivery systems in a broad field of biomedical contexts.

## Stem cell types in wound healing

To obtain optimal outcomes of wound healing, it is necessary to choose an effective stem cell source. Stem cells could be mainly divided into embryonic stem cells (ESCs), adult stem cells (ASCs), and induced pluripotent stem cells (iPSCs). Among these, ESCs and iPSCs have higher differentiation potential [15]. Furthermore, ASCs are composed of a variety of stem cells including mesenchymal stem cells (MSCs) and umbilical cord stem cells.

### Embryonic stem cells (ESCs)

ESCs are obtained from the blastocyst and characterized by pluripotent differentiation capability to three germ layers. Accordingly, there are many limitations before applying ESCs in medicine such as immunogenicity, tumorigenicity, invasive harvesting method, and especially ethical dilemmas and regulatory issues [6].

### Mesenchymal stem cells (MSCs)

MSCs, a type of ASCs, have been used more widely for wound healing. MSCs could exist in many tissues including bone marrow-derived (BM), adipose tissue-derived (AD), and fetal tissues-derived (Wharton’s jelly (WJ), umbilical cord, and placenta) MSCs [16]. A previous study investigated the functions of four kinds of MSCs (BM, AD, WJ, and placenta). The most significant T-cell inhibition was shown in WJ-MSCs, which might be used in immunosuppressive action [16]. Another study indicated that BM and placental MSCs were the appropriate cells to enhance angiogenesis with the upregulated expressions of angiogenic genes [17]. Accordingly, no single type of MSCs could be optimal for skin regeneration and wound healing. Based on specific requirements, it is hard to determine and select the most appropriate MSCs. Moreover, the invasive obtainment and unsatisfactory therapeutic efficacy of MSCs have also hindered their clinical applications. Karp et al. [18] stated that homing receptors, such as C-X-C chemokine receptor type 4 (CXCR4) that is upregulated in the bone marrow and in ischemic tissues, are usually absent on the surface of culture-expanded MSCs. CXCR4 expression might be much higher in bone marrow MSCs than other types of stem cells. However, more strategies should be taken to improve the homing capacity of MSCs.

### Induced pluripotent stem cells (iPSCs)

It is well known that iPSCs are derived by reprogramming somatic cells. Moreover, iPSCs obtain unlimited self-renewal ability and function as an abundant source of autologous or donor-matched cells for treatment. Accordingly, iPSCs have more clinical application potential than other stem cell types. It is well known that iPSCs could be differentiated into endothelial cells (iPSC-ECs) with high efficiency and excellent functions. Several studies have shown that iPSC-ECs might promote angiogenesis and perfusion recovery in peripheral arterial disease [19, 20]. Another study investigated the pro-angiogenic ability of iPSC-ECs in wound healing. The results indicated that iPSC-ECs are capable of promoting angiogenesis, perfusion, and collagen deposition and accelerating wound closure in vivo [21]. Nakayama et al. [22] prepared iPSC-MSCs from human keratinocytes and further investigated the wound-healing ability of iPSC-MSCs in an immunodeficiency mice model. In their study, iPSC-MSCs could secrete type VII collagen and promote epithelialization, thereby enhancing wound healing via subcutaneous and intravenous delivery. Despite the great advantages of iPSCs, several obstacles such as tumor formation risks, abnormalities during reprogramming, and immunogenicity are warranted to be resolved. To sum up, the engraftment efficiency of iPSCs in skin tissue might be higher, but various factors need to be taken into account during clinical applications in skin tissue.

## Optimal animal models for stem cell-based therapy in wound healing

The different layers of the skin have their structures and functions. Because rodents are more accessible among animal models, it is necessary to distinguish the differences between human and rat skins in the investigation of wound healing. The structures of the epidermis, dermis, and hypodermis are similar in humans and mice. However, their thicknesses are different [2]. Humans and mice have different percentages of leukocytes, which might influence inflammatory reactions during wound healing [23]. Several other mammals such as pigs are physiologically closer to humans. Nevertheless, pigs are limited by inadequate physiological research, higher maintenance cost in a lab, and complicated surgical operations [24]. Accordingly, mice are more widely utilized for the study of skin regeneration and wound healing. More importantly, it is necessary to take the problems of immunogenicity into account. Therefore, when using stem cells such as iPSCs, the immunodeficiency mice are regarded as the optimal animal models for wound healing [22, 25].

## Stem cell-based drug delivery systems

### Stem cell membrane-coated nanoparticles

Cell membrane-coated nanovesicles (CMNCs) could overcome the disadvantages of cell therapy and serve as more effective and safer strategies than cell therapy [26]. In previous studies, red blood cells (RBCs) and platelets have been utilized to prepare CMNCs [27], which have been widely utilized in wound healing and antibacterial infection. The serious infection is mainly induced by drug-resistant bacteria and relevant toxins. Photothermal therapy has effective antibacterial effects. But some biomaterials are toxic. Chen et al. constructed an RBC membrane-coated Fe_3_O_4_ nanoparticle for treating serious infections. This nanoparticle could function as nano-sponges to absorb and eliminate toxins with photothermal effects. The RBC@Fe_3_O_4_ nanoparticles with laser irradiation showed a superior wound-healing effect in a methicillin-resistant *Staphylococcus aureus* (MRSA) wound infection mouse model [28]. The incorporation of drug delivery nanoparticles inside biomaterial scaffolds is widely used for tissue regeneration and immune modulation. But nanoparticles could induce inflammation and hinder the application of biomaterial scaffolds. The strategy of camouflaging RBC membranes on poly(lactic-co-glycolic acid) nanoparticles (PLGA NPs) could overcome short-term and long-term inflammatory responses. With the help of the natural biocompatibility of cell membranes, anti-inflammatory protection might contribute to the recruitment of stem cells to scaffolds [29]. Tedizolid phosphate (TR-701) could effectively treat gram-positive bacteria such as MRSA. RBC membrane-coated TR-701-loaded PLGA NPs were shown with excellent biocompatibility, immune escape effect, and exotoxins neutralization ability. The bacteria inhibition and wound healing efficacy of the NPs were confirmed in the MRSA-infected mice model without toxicity [30]. Peng et al. prepared a mesoporous copper silicate microsphere (CSO) core and a platelet membrane (PM) shell as an antibacterial platform. CSO@PM could target bacteria because of the formyl peptide receptors on the PM and show effective anti-bactericidal activity with photothermal therapy. More importantly, CSO@PM was confirmed to enhance wound repair effectively [31], whereas both RBC and platelet membranes are lacking in effective targeting capability to lesions [32] and enough specific modification. More importantly, the use of CMNCs derived from RBCs or platelets is also limited by the cell sources and the genetic engineering options [33]. Therefore, stem cell membranes might overcome the above obstacles and are regarded as the appropriate source for preparing CMNCs with various advantages.

**Fig. 1:**
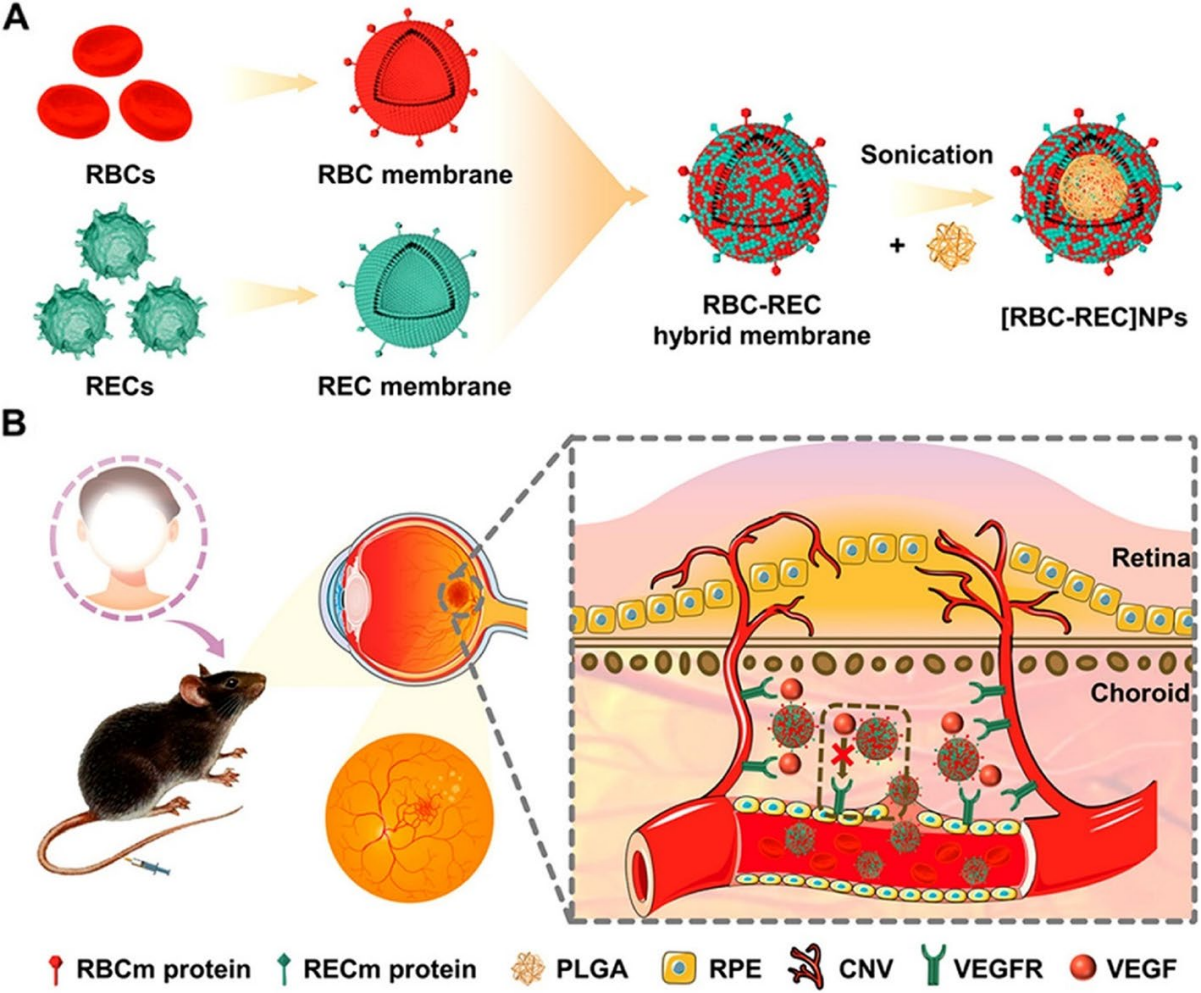
Schematic illustration of hybrid cell-membrane-cloaked biomimetic nanoparticles designed for noninvasive targeted treatment of laser-induced CNV [38]. The membranes of retinal endothelial cells (RECs) and red blood cells (RBCs) were used to prepare the hybrid cell membranes. Reproduced from Li et al. with permission from American Chemical Society, Copyright 2021

Vascular endothelial injury and tissue ischemia are important factors affecting wound healing. The peripheral vascular disease could result in remarkable morbidity and mortality in high-risk populations such as diabetes and so on [34]. Several studies have developed CMNCs to treat endothelial injury. A study prepared a nanoplatform of antimalarial drug dihydroartemisinin coated by cell membranes of brain microvascular endothelial cells (BMECs) [35]. This membrane-coated nanodrug significantly inhibited parasites residing in RBCs obstructed in the BMECs. Mechanically, this nanoplatform might obtain the self-targeting capability of endothelial cells [36, 37]. As for choroidal neovascularization (CNV), hybrid cell membrane-coated nanoparticles were developed for the noninvasively targeted treatment of CNV [38]. The membranes of retinal endothelial cells (RECs) were equipped with homologous targeting ability of blood vessels. The RBC membranes could protect the nanoparticles from phagocytosis with immune evasion ability. These hybrid NPs effectively accumulated in CNV regions and largely reduced the area and leakage of CNV, resulting in excellent therapeutic efficacy (Fig. 1).

**Fig. 2:**
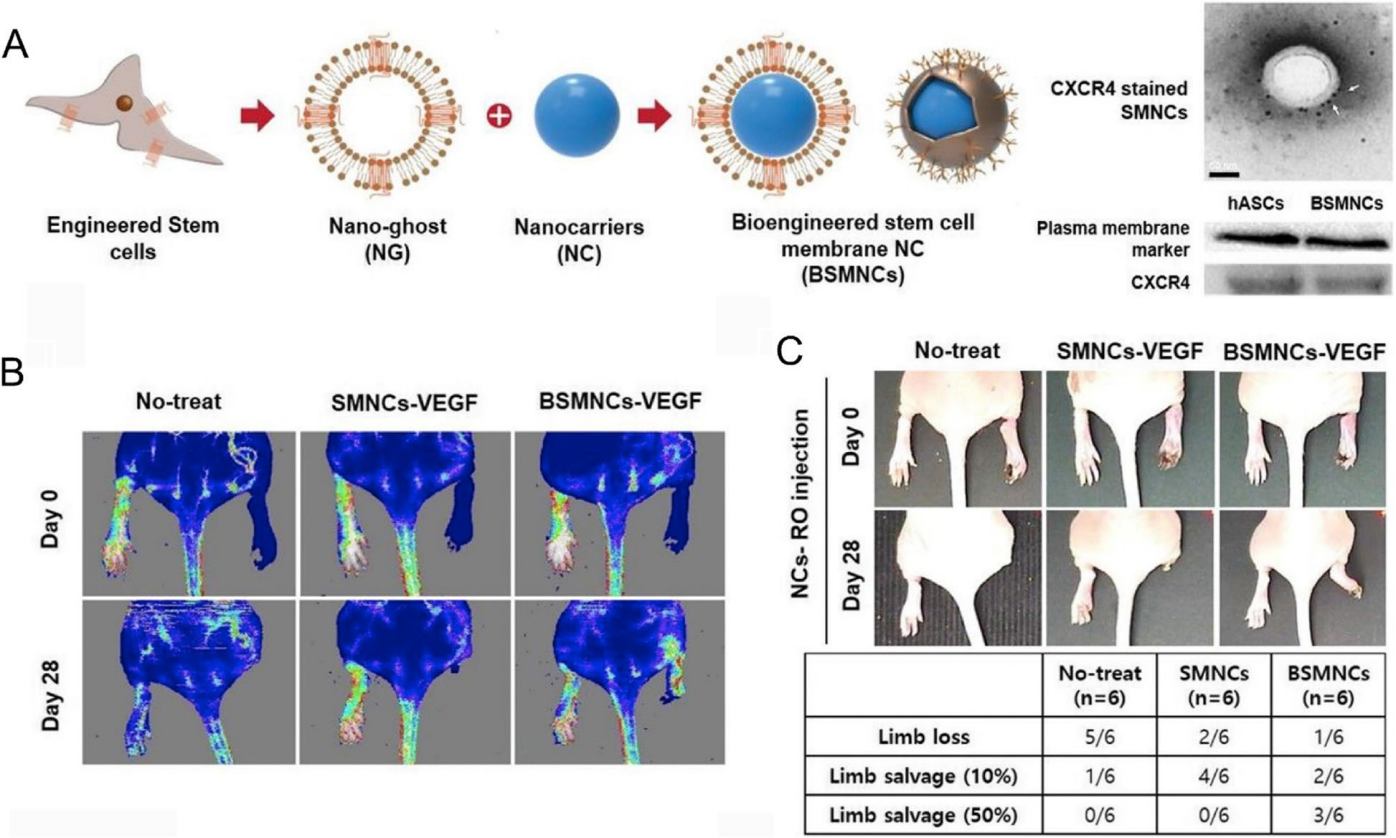
Characterization of bioengineered stem cell membrane nanocarriers (BSMNCs) and revascularization of ischemic limb and reduction of limb loss of BSMNCs and stem cell membrane nanocarriers (SMNCs) in a murine hindlimb ischemia model [45]. **A** Schematic showing the preparation of BSMNCs. **B** Representative laser Doppler perfusion imaging of hindlimb ischemia treated with SMNCs or VEGF-BSMNCs. **C** Physiological status of ischemic hind limb before and 28 days after injection. Reproduced from Bose et al. with permission from Elsevier Ltd., Copyright 2018

Engineered stem cell therapy is widely investigated owing to the excellent functions of stem cells [39], whereas the application of stem cells for peripheral vascular disease is limited owing to safety, scalability, and reproducibility. Based on this, stem cell membrane-coating nanotechnology has been utilized to resolve the above problems in wound healing and vascular disease [27, 33]. It has been confirmed that the ischemic tissue-directed stem cell homing ability mainly relies on the interactions between CXCR4 on stem cell membranes and stromal-derived factor (SDF) secreted by injured lesions [40]. Chemokine and cytokine receptors including CXCR1/2 and CCR1/2 are highly expressed on stem cell membranes, which contribute to stem cell migration toward inflammatory or injury lesions [41]. One study reported that overexpression of CXCR4 and CXCR12 could result in stem cells targeting inflammatory and ischemic sites [42]. Numerous studies have bioengineered stem cells to enhance their functions by expressing some novel molecules. Moreover, several genetic engineering methods are utilized to enhance stem cell homing to injury lesions [43, 44]. Stem cells are characterized by their prominent properties of low immunogenicity and immunomodulation and might function as a promising therapeutic tool. For example, one study prepared engineered stem cell membrane-coated nanovesicles for enhancing targeted delivery to ischemic hindlimbs [45]. Based on the CXCR4-induced homing mechanisms, the adipose-derived stem cells were designed to overexpress CXCR4 and utilized for coating VEGF-loaded nanocarriers, which showed effective limb recovery in ischemic tissues (Fig. 2). Stem cell membranes significantly reduced the immune clearance of NPs and enhanced penetration across inflamed endothelial layers.

Taken together, stem cell membrane-coated nanovesicles have the following advantages: (a) stem cell membrane-coated nanovesicles could contribute to targeting ischemic lesions, thereby promoting blood perfusion and limb salvage, (b) stem cell membrane coating significantly reduces the uptake by immune cells with the ability to immune escape, and (c) stem cell membrane might also enhance the translocation across endothelial barriers. Because of the benefits of scalable stem cell sources, stem cell membrane-coated nanovesicles are supposed to have tremendous potential for targeted drug delivery in the treatment of skin regeneration and wound healing.

### Stem cell-derived extracellular vesicles

**Fig. 3:**
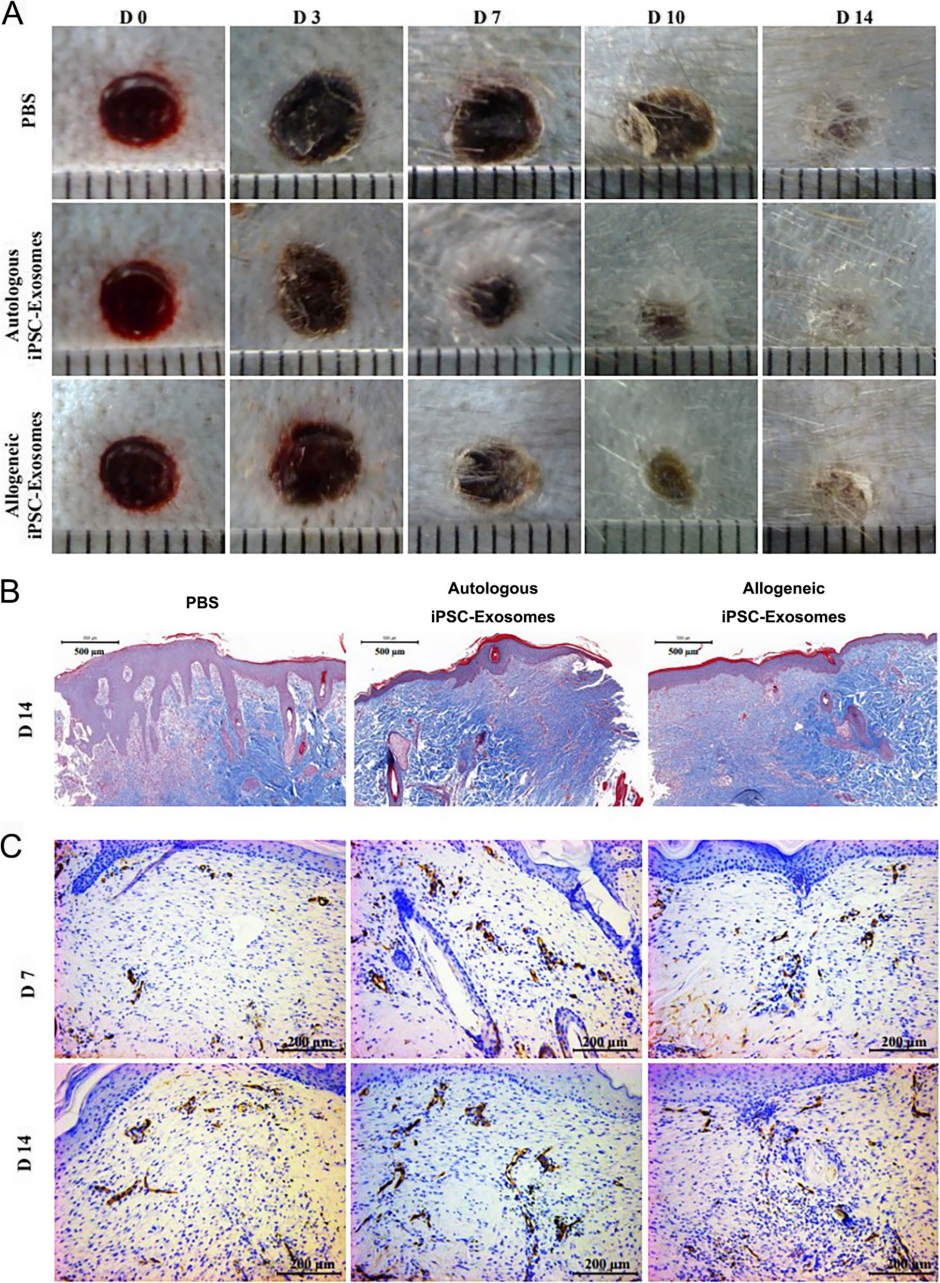
Macaque iPSC-derived exosomes promote wound healing and angiogenesis [50]. **A** Representative images of wounds treated with PBS, autologous, and allogeneic iPSC exosomes. **B** Representative images of epithelial coverage and collagen deposition in the wounds. **C** Representative images of wound sections stained for CD34. Reproduced from Lu et al. with permission from Elsevier B. V., Copyright 2019

Stem cell-derived extracellular vesicles might be another effective cell-free therapy rather than cell-based therapy. It has been confirmed that stem cells play a vital role in tissue regeneration via paracrine abilities rather than differentiation [6]. The cytoplasm membrane and the multivesicular bodies formed by invagination could fuse to secret exosomes. Exosomes could transfer functional molecules including various RNAs and proteins and promote the communication among cells and the mediation of paracrine [46, 47]. The therapeutic roles of exosomes gradually obtain a wide interest in promoting tissue repair and regeneration. Zhang et al. [11] investigated the role of exosomes derived from induced pluripotent stem cell-derived mesenchymal stem cells (iPSC-MSCs) in treating cutaneous wounds. In their study, iPSC-MSC exosomes might promote reepithelialization and collagen maturity and decrease scar widths. Another study showed that miRNAs in exosomes derived from MSCs might promote the function of skin fibroblasts [48]. Moreover, MSCs-derived exosomes with upregulated miRNA-181c might inhibit the Toll-like receptor 4 pathway, thereby modulating inflammation in burn injury [49]. Lu et al. [50] aimed to detect the effects of autologous iPSCs and exosomes in wound healing. In their study, all of the autologous and allogeneic iPSCs and exosomes could promote wound healing (Fig. 3). However, allogeneic iPSC exosomes are recommended to be the preferred choice because of the mass production and no risk of teratoma formation. Nevertheless, the therapeutic molecules in exosomes and the underlying mechanisms should be further investigated.

Cell-free therapy is a promising strategy in tissue repair and regeneration by replacing stem cells themselves with exosomes. The main advantages include overcoming poor cell engraftment and reducing immune rejection in cell-based therapy. More importantly, exosomes could be stored safely and easily when compared with stem cells, which is important to cut down the cost. Taken together, similar to the stem cell membrane-coated nanovesicles, exosomes could obtain the functions of their parent cells and deliver therapeutic agents.

### Stem cells as drug carriers

**Fig. 4:**
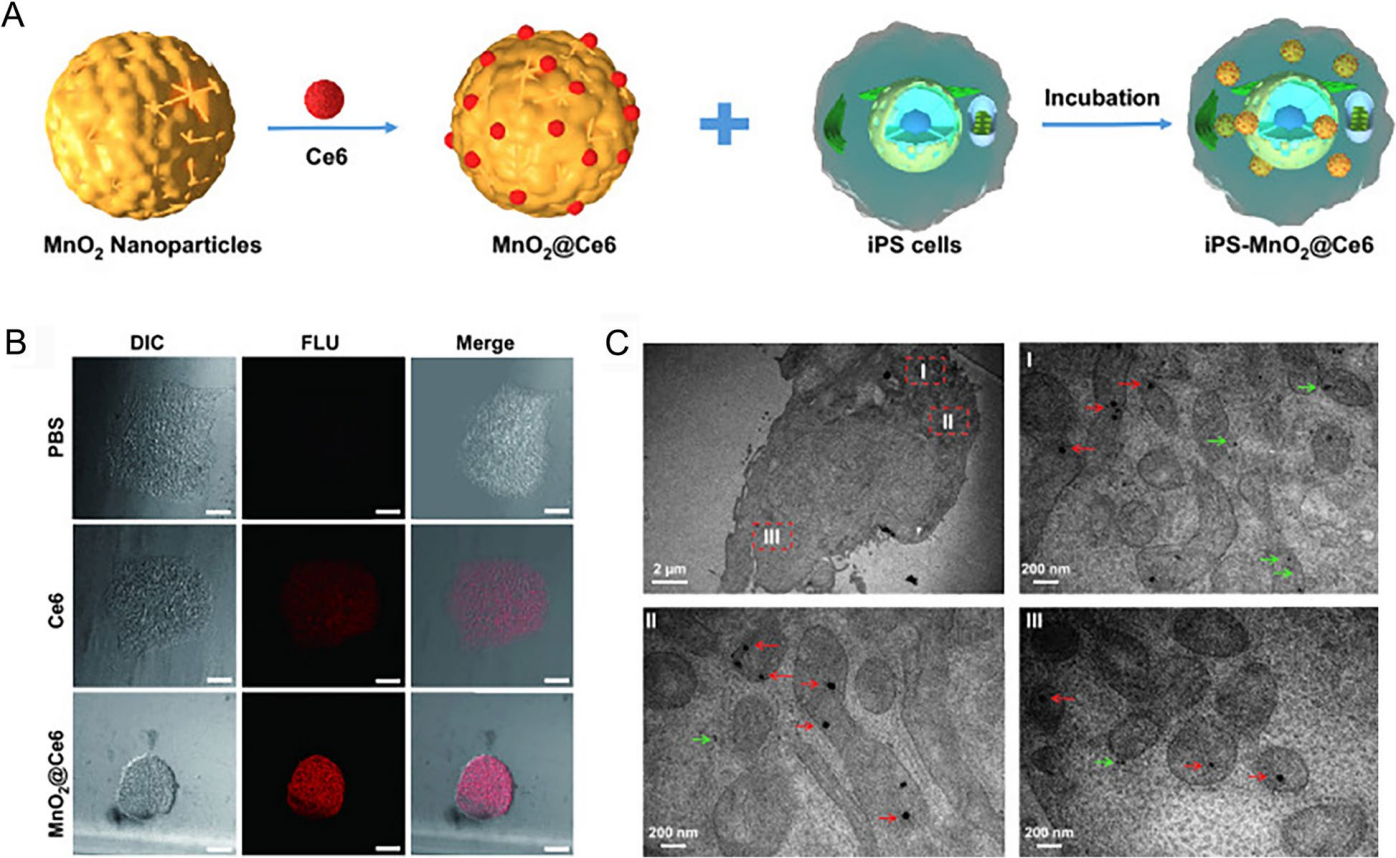
Preparation and characteristics of iPS-MnO_2_@Ce6 as drug carriers [12]. A Schematic representation of the preparation process of iPS-MnO2@Ce6. **B** Confocal laser scanning microscopy images of iPSCs treated with Ce6 and MnO_2_@Ce6. **C** Representative transmission electron microscope images of iPSCs incubated with nanoprobes. The red arrows indicate the nanoprobe without degradation, and the green indicates the nanoprobe with degradation. Reproduced from Liu et al. with permission from Springer, Copyright 2020

Compared with MSCs, iPSCs have attracted growing interest because of easily generated by reprogramming differentiated somatic cells with just transcription factors [51]. Accordingly, iPSCs might serve as drug delivery carriers in various diseases. Recently, previous study has investigated the feasibility of using iPSC [52] as carriers for anti-tumor drugs. The results showed that iPSCs could effectively target tumor tissues and further be killed by the photothermal effects induced by gold NPs [52]. Mitomycin-treated iPSCs could also perform excellent tumor-targeting capability [53] and were used to deliver MnO_2_@Ce6 nanoparticles into tumors for synthetic photodynamic and immunotherapy [12]. In their study, the MnO_2_@Ce6-loaded iPSCs could target tumors in vivo and contribute to anti-tumor immune response efficiently (Fig. 4). Based on the above studies, stem cells as drug carriers are regarded to have tremendous potential for skin regeneration and wound healing based on targeted delivery.

However, as shown in other studies, iPSCs might distribute in other normal tissues [54], which induces side effects because of these residual stem cells. Accordingly, it is supposed that when stem cells as drug carriers have targeted and accumulated in lesions, stem cells must further die in the entire body after the therapeutic effects. To improve the side effect of iPSCs, engineered neural stem cells derived from iPSCs were utilized to exhibit therapeutic effects in the tumor, which showed no toxicity to normal nontargeted organs [55]. Taken together, despite the great advantages of iPSCs compared with MSCs, the safety of iPSCs in skin regeneration and wound healing should be further investigated in future studies.

### Scaffold-free stem cell sheets

Scaffold-free skin equivalents could utilize cellular functions to mimic native skin tissue [13]. Cell sheet technology serves as an important scaffold-free method because of the simple preparation process [56], good fusion with native skin tissue [57], and the possibility of fabricating from the patient’s cells [58]. Cell sheets form two- or three-dimensional geometry in appropriate conditions without scaffolds. Different cell types such as skin keratinocytes, fibroblasts, and stem cells are grown on a plastic culture plate to mimic their naive structure [59], whereas there are several technical obstacles to using scaffold-free cell sheets including a prolonged culture period, limited volume for implantation, inherent physical weakness, and poor vascularization [60]. Accordingly, engineering technologies such as hydrogels and scaffolds should be applied to resolve these disadvantages of scaffold-free cell sheets [61].

### Stem cell-laden scaffolds

During the delivery of stem cells in wound sites, the survival and function of cells are of great importance in ensuring cell efficacy [6]. Local injection and intravenous infusion have their disadvantages. For example, the local injection might influence the cell membrane integrity because of the mechanical stresses induced by the needle. Although intravenous infusion could be less invasive, the number of stem cells accumulating in wound sites might be limited [62]. Accordingly, stem cell-laden scaffolds are necessary to be prepared to improve stem cell survival, thereby promoting transplantation efficiency.

Hydrogels are one of the most common scaffolds that could safely load cells and biological factors. Most importantly, hydrogels could be used in defective skin sites and are degradable to promote skin repair [63]. Various hydrogels are commonly used to deliver stem cells for skin regeneration and wound healing [64, 65]. Many types of cells such as stem cells are utilized to prepare stem cell-enabled biomimetic ECM structure and skin regeneration [66, 67]. Stem cells in hydrogels have the following functions: (a) promoting wound healing by secreting a variety of growth factors such as TGF-β and (b) differentiating into specific cell types involved in wound repair including keratinocytes, fibroblasts, and endothelial cells. Taken together, stem cells in hydrogels could promote wound healing by enhancing vascularization and reepithelialization and reducing granulation formation [68, 69].

Compared with single hydrogel, the bilayer nanofiber/hydrogel hybrid skin grafts are capable of mimicking the skin structure of the epidermis and dermis and the biological and mechanical functions. One study constructed a hybrid made of PLCL/poloxamer nanofiber and dextran/gelatin hydrogel for skin repair [14]. The hybrid scaffolds showed improved mechanical functions and enhanced proliferation and differentiation of adipose-derived stem cells. This kind of hybrid scaffold might have the potential to be a temporary skin replacement.

## Conclusions and future outlooks

Among the three common drug delivery systems including stem cell membrane-coated NPs, stem cell-derived extracellular vesicles, and stem cell as drug carriers, they have their advantages and disadvantages (Table 1). The applications of stem cell-derived extracellular vesicles are hindered by the high cost and difficult purification process. As for stem cells as drug carriers, the safety in vivo should be further resolved. For instance, Yamanaka et al. [70] and Zhong et al. [71] try to diminish the tumorigenesis risk of iPSC technology, including optimizing the cocktail of reprogramming factors, using the strategy of chemical inductive reprogramming, controlling mutagenesis of host gene caused by retroviral insertion via exogenous DNA-free vectors, eliminating aberrant cells via drug-inducible suicide system, and increasing purity of iPSC samples. Stem cell membrane-coating nanotechnology is regarded as a novel drug delivery system with the great advantages of both natural and synthetic components. As a biological component, stem cell membranes obtain specific surface properties including targeting capability to injure lesions. Furthermore, the core NPs could load drugs for different purposes. With these promising functions, stem cell membrane-coated NPs have greater potential for clinical applications than other stem cell-based drug delivery systems, whereas further improvement of stem cell membrane-coating technology is still necessary. The ligands on the surface of stem cell membranes are of great importance in the targeting and homing abilities of stem cells. Compared with the traditional extrusion or sonication technologies for cell membrane coating, a novel approach via microfluidic and electroporation technology [72] could contribute to improved membrane-coating processes and membrane functions.

**Table 1:** The summary of stem cell-based drug delivery strategy for skin regeneration and wound healing

Strategy	Advantages	Disadvantages
**Stem cell membrane-coated nanoparticles**	Targeting ischemic lesions	Insufficient product quality
	Reducing the uptake by immune cells with the ability to immune escape	
	Enhancing the translocation across endothelial barriers	
	Reducing the risk of teratoma formation	
	Easy to the mass production	
	Lower cost when compared with preparing extracellular vesicles	
**Stem cell-derived extracellular vesicles**	Overcoming poor cell engraftment and reducing immune rejection in cell-based therapy	Hard to achieve the mass production
	Extracellular vesicles could be stored safely and easily	Higher cost
	Extracellular vesicles could obtain the functions of their parent cells and deliver therapeutic agents	
	No risk of teratoma formation	
**Stem cells as drug carriers**	iPSCs could effectively target injured tissues	iPSCs might distribute in other normal tissues, which induces side effects
		The risk of teratoma formation
**Scaffold-free stem cell sheets**	The simple preparation process, good fusion with native skin tissue, the possibility of fabricating from the patient’s cells	A prolonged culture period, limited volume for implantation, inherent physical weakness, and poor vascularization
**Stem cell-laden scaffolds**	Improving stem cell survival and promoting transplantation efficiency	The risk of teratoma formation
	Mimicking the skin structure of the epidermis and dermis	

The complexity and heterogeneity of stem cell membranes have novel advantages over other cell membranes. However, it should not be ignored that cell membrane nanotechnology is still at an early stage, and it remains to be further explored. For example, the hybrid cell membranes could obtain different functions from a variety of cell membrane sources such as RBCs and bacteria that express self-recognition molecules. In this way, NPs coated with hybrids of the stem cell membrane and other cells could be characterized by longer retention time, better inflammation targeting, and excellent immune evasion, which is deemed to be a promising drug delivery system shortly. Moreover, it is necessary to apply cell membrane-coating nanobiotechnology in other areas. Zhang et al. coated beta-cell membranes onto electrospun nanofibrous scaffolds to promote cell survival and tissue regeneration [73]. Accordingly, cell membrane-coating nanobiotechnology is capable of changing the surface properties of scaffolds to promote tissue regeneration, which might be further utilized in stem cell-laden scaffolds for wound healing.

The recent development of 3D bioprinting technology might offer architectural organization of native skin precisely, whereas more bioactive bioinks are warranted to be prepared to construct 3D-bioprinted constructs in the application of wound healing. Furthermore, cells such as fibroblasts, epidermal progenitors, and endothelial cells are combined with optimal bioinks to mimic the skin-specific microenvironment, thereby promoting functional recapitulation of native skin. More interestingly, we believe that a handheld 3D bioprinter might be developed to conveniently apply a stem cell-laden scaffold to the wounds especially in operation room [65]. One previous review of Ojeh et al. [7] concluded some of the ongoing or completed clinical trials registered on www.clinicaltrials.gov. In these clinical trials, stem cells can be delivered to the skin wounds either directly or via skin scaffolds. Accordingly, the preliminary exploration of clinical application using stem cell-based drug delivery strategy in the area of skin regeneration and wound healing has begun.

**Fig. 5:**
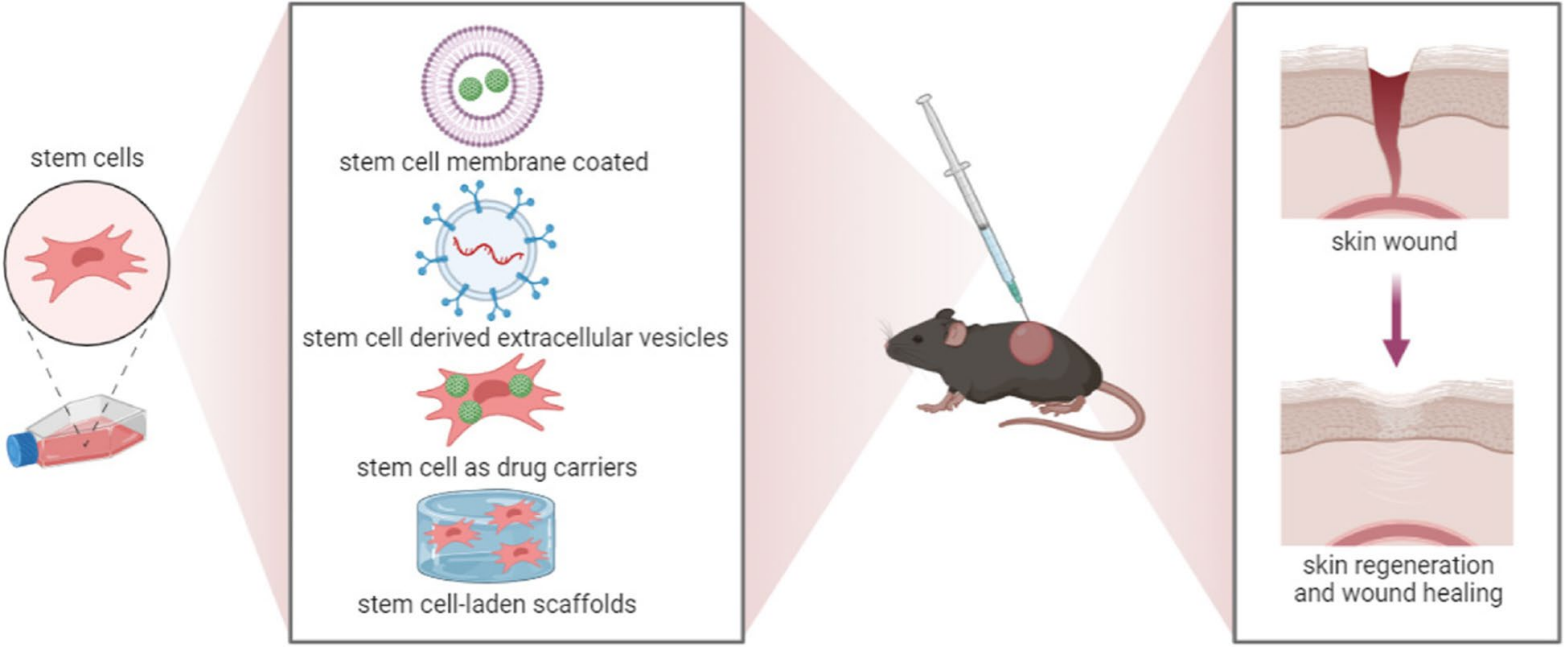
Stem cell-based drug delivery strategy for skin regeneration and wound healing: potential clinical applications. Stem cell-based drug delivery strategies include stem cell membrane-coated NPs, stem cell-derived extracellular vesicles, stem cell as drug carriers, and stem cell-laden scaffolds. Stem cell-based drug delivery systems hold considerable promise as novel strategies for the treatment of skin regeneration and wound healing

Taken together, stem cell-based drug delivery systems hold considerable promise as novel strategies for the treatment of skin regeneration and wound healing. Especially, stem cell membrane-coating nanotechnology confers great advantages compared to other drug delivery systems and might be utilized in a broad field of biomedical contexts (Fig. 5).

## Data Availability

All data generated or analyzed during this study are available from the corresponding author.
